# A hook wire dislodged into the subglottic area as a rare complication following computed tomography-guided hook wire localization: a case report

**DOI:** 10.1186/s12890-022-02065-0

**Published:** 2022-08-17

**Authors:** Haiyin Fan, Changying Guo, Bin Zou

**Affiliations:** Department of Thoracic Surgery, Jiangxi Cancer Hospital, No. 519, Beijing East Road, Nanchang, 330029 China

**Keywords:** Pulmonary nodules, Hook wire, Video-assisted thoracic surgery, Case report

## Abstract

**Background:**

Computed tomography-guided hook wire localization (CT-GHWL) was used to localize the small pulmonary nodules before video-assisted thoracic surgery (VATS). Its associated complications included hook wire dislodgement, pulmonary hemorrhage, and pneumothorax. This is the first report of a patient with a hook wire sliding into the subglottic area after CT-GHWL.

**Case presentation:**

A 27-year-old female had productive cough for 8 days. A high-resolution CT scan showed a 12 mm part-solid nodule in the number 8 segment of the left lung. Prior to VATS, she received CT-GHWL to localize the nodule. During VATS, the hook wire unexpectedly slid away. A chest computed tomography was immediately performed and the sagittal reconstructed images showed the needle at the subglottic area. Finally, the needle was extracted by biopsy forceps under bronchoscope evaluation. The patient was eventually recovered and discharged.

**Conclusions:**

Dislodge of the hook wire into the subglottic area is an extremely rare but serious complication following CT-GHWL. Attention should be paid to securing the needle on the lung surface during VATS.

## Background

With the advent of computed tomography screening for lung cancer, an increasing number of patients have been found to have small and undetermined pulmonary nodules, especially ground-glass opacity nodules (GGNs) [1]. Video-assisted thoracic surgery (VATS) provides a minimally invasive way to diagnose and manage these pulmonary nodules [2]. However, during VATS, it is often difficult to localize small nodules, especially subsolid or deep nodules [3]. Computed tomography-guided hook wire localization (CT-GHWL) is able to accurately detect these nodules [4]. Commonly reported complications of CT-GHWL include hook wire dislodgement, pulmonary hemorrhage, and pneumothorax [5]. This is the first report of a patient with the hook wire dislodged into the subglottic area.

## Case presentation

**Fig. 1:**
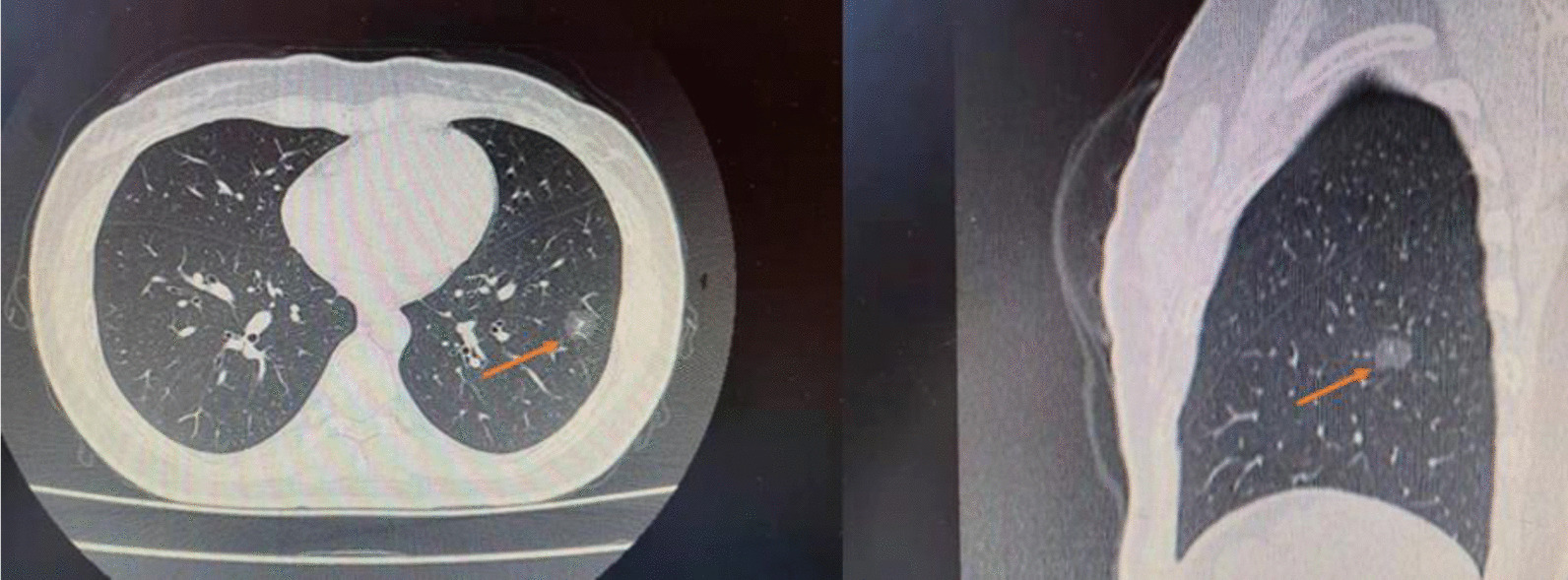
Computed tomography showed a 12 mm mixed ground glass opacity nodule (arrow) that was centrally located in the number 8 segment of the left lung (Left, axial plane; Right, sagittal plane)

**Fig. 2:**
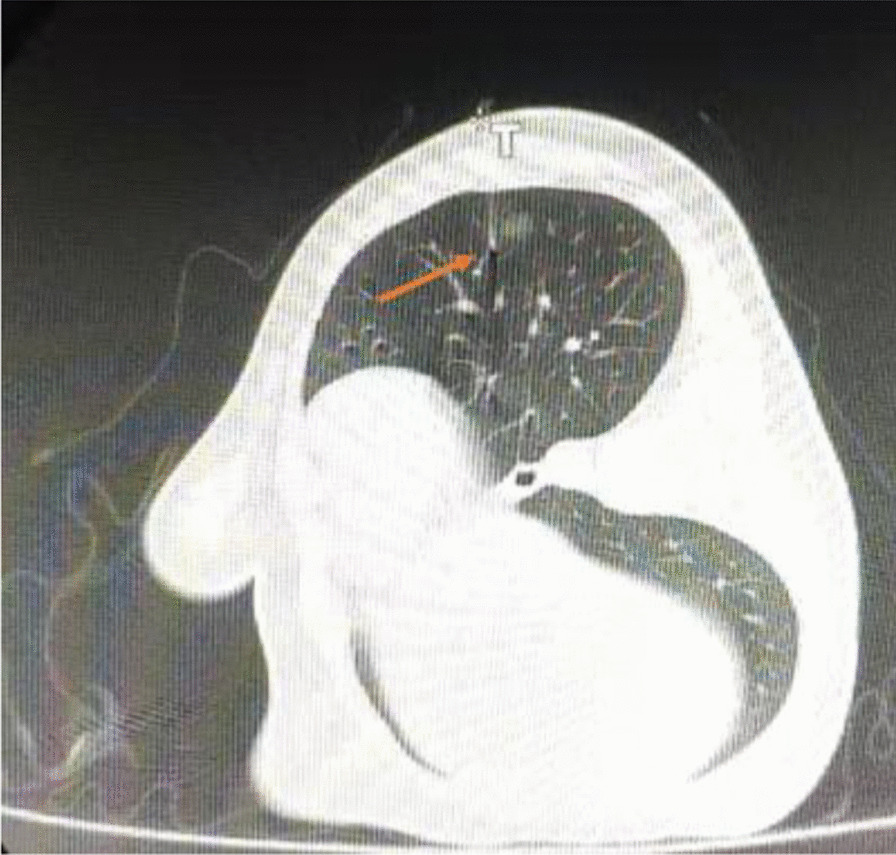
Computed tomography was performed again to ensure the wire location. The hook-shaped wire tip (arrow) was secured at a distance of 1.0 cm from the nodule

A 27-year-old female had productive cough for 8 days, with no response to the levofloxacin treatment. A high-resolution CT scan showed a 12 mm part-solid nodule (consolidation to tumor ratio 0.17) that was located centrally in the number 8 segment of left lung (LS8) (Fig. 1). She was scheduled for CT-GHWL to ensure an adequate surgical margin from the nodule prior to VATS segmentectomy of LS8. After determining the puncture site by initial CT scan and applying local anesthesia, a hook wire was entered through a 20 gauge needle (Mammography Localization Set; PAJUNK, Geisingen, Germany). Another CT scan was performed to ensure the wire location. The tip of the hook-shaped wire was secured at a distance of 1.0 cm from the nodule (Fig. 2). There was no obvious hemorrhage and pneumothorax after the procedure.

**Fig. 3:**
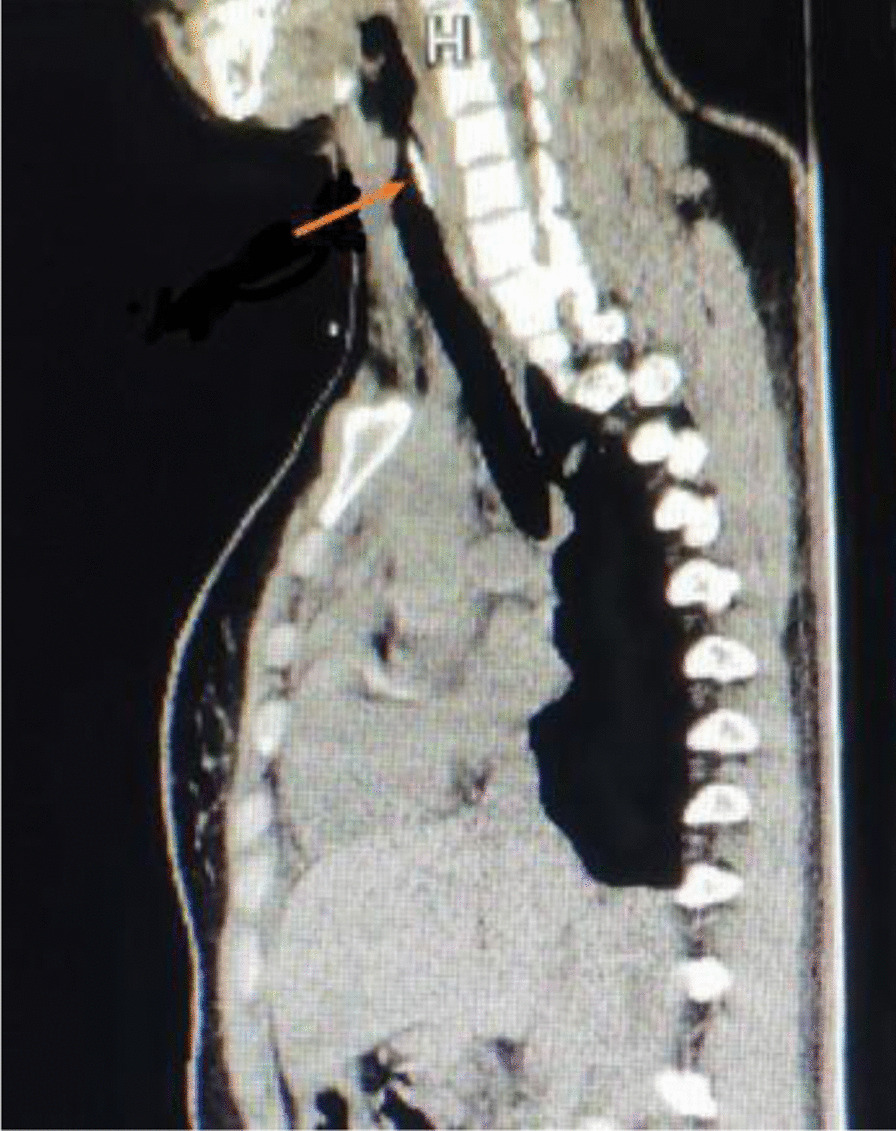
Sagittal reconstruction on the computed tomography images showed a metal spot (arrow) in the subglottic area

After double lumen endotracheal intubation under general anesthesia, the operation was performed on segmentectomy of LS8 through a single-port VATS. When entering the thoracic cavity, the hook wire was visualized in the left lower lung lobe. Its tail outside the chest wall was resected to minimize any bacterial contamination. After the lower pulmonary ligament was freed, the needle suddenly disappeared in the video-assisted thoracoscopic vision field. The hook wire was not found after the extensive search of the left thoracic cavity. The needle was also not found in the lesion or residual lung after resection of LS8. Bronchoscopy did not find any foreign body in the left bronchus. We immediately performed a chest X-ray examination, which also did not identify the needle. Therefore, the skin incision was lengthened to approximately 8 cm, with no hook wire found by manual palpation of the residual left lung. The chest X-ray examination was performed again after the patient turned to the supine position. A high-density shadow was observed near the left main bronchus, which could not be differentiated from the metal in the intubation tube. A chest CT scan was done after removing the tracheal intubation tube. We performed sagittal reconstruction on the CT images. It showed a metal spot in the subglottic area, which was considered the “wandering” hook wire (Fig. 3). The patient was transferred to the tracheoscopic interventional unit. The front-end hook wire was removed by biopsy forceps under the flexible bronchoscopy. The patient recovered well and was discharged 1 week after the operation. Postoperative pathology showed adenocarcinoma, mainly the adherent growth type.

## Discussion and conclusions

Peripheral pulmonary nodules, such as GGNs, are commonly difficult to palpate and cannot be found by the naked eyes during thoracoscopic resection, which affects the VATS success ratio, and even leads to a higher requirement for open chest surgeries. The CT-GHWL before VATS is simple and easy to use [6]. Most complications of CT-GHWL include pneumothorax, hemothorax, pulmonary hemorrhage, aeroembolism, and hook wire dislodgement. Here, we reported a hook wire dislodged into the subglottic area, which, to the best of our knowledge, has not been previously reported.

We consider the following possible causes for the hook wire swimming. 1) The hook wire entered the small bronchus during the process of CT positioning. During the VATS operation, when the lung was manipulated repeatedly, the hook wire could only move forward due to the barb on its tip, resulting in hook wire swimming. 2) Once the operator found that the needle was missing, he repeatedly palpated the main bronchus by hand to search for the needle, which could have squeezed the needle into the main bronchus, resulting in hook wire swimming. 3) When the endotracheal tube was removed from the patient, the endotracheal tube brought the hook wire from the left main bronchus to the subglottic area. Because the glottis was relatively narrow, the hook wire was stuck under the glottis.

In our patient, the design of the mammography localization set might be the reason for the unexpected hook wire slide away into the lung. Previous studies also reported the slides of the similar hook wire into the left upper lobe pulmonary artery or the splenic artery [7, 8]. Another spiral-shaped hook wire, the Lung Marker System (Somatex Lung Marker System®, Berlin, Germany) that is specifically designed for the CT-guided marking of pulmonary nodules, can increase the contact area and friction of the wire with the pulmonary parenchyma, making it extremely reliable and secure during the procedure [9].

In conclusion, we reported the first case of the hook wire sliding into the subglottic area during CT-GHWL. The following lessons were learned from this case study. When the hook wire is found on the lung surface during VATS, it should be immediately secured on the lung surface with the surgical forceps or hemolock to prevent dislodge of the guide wire. Once the hook wire disappears from the surgical field, a CT examination should be performed immediately to look for the needle. Every effort should be attempted to localize and remove the dislodged needle.

## Data Availability

Data sharing is not applicable to this article as no datasets were generated or analyzed during the current study**.**

## Abbreviations

CT-GHWL: Computed tomography-guided hook wire localization
VATS: Video-assisted thoracic surgery
CT: Computed tomography
GGNs: Ground-glass opacity nodules
